# Factors Influencing COVID-19 Vaccine Hesitancy in Pregnant and Breastfeeding/Puerperium Women: A Cross-Sectional Study

**DOI:** 10.3390/vaccines12070772

**Published:** 2024-07-14

**Authors:** Dania Comparcini, Marco Tomietto, Francesco Pastore, Bethany Nichol, Daniela Miniscalco, Maria Elena Flacco, Pasquale Stefanizzi, Silvio Tafuri, Giancarlo Cicolini, Valentina Simonetti

**Affiliations:** 1 Interdisciplinary Department of Medicine, “Aldo Moro”, University of Bari, 70121 Bari, Italy; pasquale.stefanizzi@uniba.it (P.S.); silvio.tafuri@uniba.it (S.T.); 2 Department of Nursing, Midwifery and Health, Faculty of Health and Life Sciences, Northumbria University, Newcastle upon Tyne NE1 8ST, UK; marco.tomietto@northumbria.ac.uk; 3 Department of Biomedicine and Prevention, TorVergata University, 00133 Roma, Italy; francesco.pastore@uniba.it; 4 Department of Social Work, Education and Community Wellbeing, Faculty of Health and Life Sciences, Northumbria University, Newcastle upon Tyne NE1 8ST, UK; bethany.nichol@northumbria.ac.uk; 5 “A. Costa” Hospital, 40046 Bologna, Italy; minis.dany@gmail.com; 6 Department of Environmental and Prevention Sciences, University of Ferrara, 44121 Ferrara, Italy; mariaelena.flacco@unife.it; 7 Department of Innovative Technologies in Medicine and Dentistry, “Gabriele D’Annunzio” University of Chieti, 66100 Chieti, Italy; g.cicolini@unich.it (G.C.); v.simonetti@unich.it (V.S.)

**Keywords:** COVID-19, vaccination hesitancy, vaccination attitude, pregnancy, breastfeeding

## Abstract

Vaccination among pregnant and breastfeeding women is critical for protecting this vulnerable population and their children. COVID-19 vaccination is recommended both during pregnancy and breastfeeding; however, we still do not fully understand the determinants that influence hesitancy towards COVID-19 vaccination. This study aimed to identify the determinants of vaccine hesitancy in pregnant and breastfeeding, puerperium women. A multicenter, cross-sectional study, involving 435 pregnant and breastfeeding women, was conducted. Vaccination hesitancy was evaluated by administering the Vaccination Attitudes (VAX) Scale and the Zung Anxiety Self-Assessment Scale (SAS) was adopted to measure anxiety levels. Overall, 14% of the participants reported that they did not receive the COVID-19 vaccine, and 78.3% received their first dose during pregnancy or while breastfeeding. The descriptive statistics for the VAX scale showed a total mean score of 3.35 (±1.6), and 75% of participants reported an anxiety index equal to or lower than the threshold. Vaccine hesitancy increased as “adverse events after vaccination” increased (*p* < 0.01), while SAS levels positively correlated with the participants’ mean age (*p* < 0.05). Investigating the factors influencing vaccine hesitancy enables the development of targeted health policies and SARS-CoV-2 vaccination programs.

## 1. Introduction

The COVID-19 pandemic highlighted the importance of being prepared for major public health threats and the importance of improving social attitudes and behaviors such as vaccination hesitancy, namely the refusal or delay to accept vaccination despite sufficient availability [1]. In particular, vaccination amongst pregnant and breastfeeding women is imperative for protecting this vulnerable population [2] and their children [3]. During the first phase of COVID-19 vaccination delivery in December 2020, pregnant women were excluded from the high-priority risk grouping of vulnerable populations [4] despite evidence suggesting they are at a higher risk of morbidity and mortality from COVID-19 when compared with non-pregnant women of reproductive age [5] and are more susceptible to severe COVID-19 symptoms including admission to intensive care [6]. COVID-19 in pregnancy has also been associated with an increased stillbirth rate [7]. In addition, pregnant women were excluded from phases II and III of clinical trials on the efficacy and safety of COVID-19 vaccines [8]. Nonetheless, the existing data suggest the same effectiveness of COVID-19 vaccination between pregnant and non-pregnant individuals [9] and the findings from recent studies support the safety of COVID-19 vaccination during pregnancy [10]. Currently, COVID-19 vaccination is recommended both during pregnancy and while breastfeeding [11] to protect groups at high risk of severe COVID-19. However, when COVID-19 vaccination programs started in Europe on 27 December 2020 with the “vaccine day” [12], evidence to inform decision-making on vaccination during pregnancy and while breastfeeding was lacking due to the small amount of evidence available. Countries around the world adopted different approaches to COVID-19 vaccination to prioritize the vaccination of health workers and at-risk groups to stop severe disease and death, keep health workers safe, and reopen societies and economies [13]. In Italy, from October 2022, considering the new available COVID-19 vaccines, the previous indications were further updated, introducing a second booster dose (fourth dose) for pregnant and breastfeeding women [14].

Achieving good vaccination coverage among pregnant and breastfeeding women remains a global challenge. In fact, during the 2022–2023 influenza season, only 27.3% of women received a COVID-19 bivalent booster vaccine before or during pregnancy [15]. Indeed, vaccination campaigns to address vaccine hesitancy, especially if tailored explicitly by population group, are a cost-effective solution to increase vaccination rates [16] and to increase public confidence in governments and vaccines [17]. To date, the identified determinants of hesitancy towards COVID-19 vaccination include the evolving pandemic and vaccines, the emergence of new variants and strains, lack of information/misinformation circulating on social media, fake news, anti-vaccine movements, development of vaccines in a fast manner, and suspicion of political intervention [18], reducing the perception of the efficacy and safety of new vaccines. In addition, in pregnant women, a lack of knowledge and awareness regarding the vaccine’s benefits and necessity, little trust in the safety and effectiveness of the vaccine, and poor involvement of health workers in promoting vaccination contribute to vaccine hesitancy [19]. Understanding the reasons for vaccination hesitancy and low coverage in pregnancy (which can be linked to the individual woman, the vaccinator, policies, or structural factors) is crucial for achieving higher COVID-19 vaccination acceptance and coverage in pregnant women [20].

Vaccine-hesitant individuals may accept all vaccines but remain concerned about vaccine safety; some may refuse or delay some vaccines but accept others; some individuals may refuse all vaccines because of a lack of trust in the government or the healthcare system and suspicion of profiteering by pharmaceutical companies [21]. The WHO-SAGE “Model of Determinants of Vaccine Hesitancy” [22] classified vaccine hesitancy factors into three domains: (1) contextual influences (historical, sociocultural, environmental, health system/institutional, economic, or political factors); (2) individual and group influences (personal perception of the vaccine or influence from the social/peer environment); and (3) vaccine and vaccination-specific issues (related to the characteristics of the vaccine or the vaccination process). In particular, several factors influence vaccine hesitancy: (1) confidence (do not trust the vaccine or provider), (2) complacency (do not value the vaccine or perceive a need for it), and (3) convenience (challenges around access), which place the individual on a continuum of indecision between refusing and accepting all vaccinations.

Previous studies showed that information-seeking behavior was associated with higher anxiety levels [23], especially during periods of high risk for health status [24] or health threats such as during the COVID-19 pandemic [25]. In this context, the individual overestimation of perceived threats [26] and the related greater intolerance to uncertainty result in difficulty in making decisions [27]. However, it is well established that for all recommended vaccinations for pregnant women, an altered perception of risk as well as the lack of specific information provided by healthcare professionals are associated with a low vaccination coverage [28]. In this sense, higher levels of anxiety could be associated with greater COVID-19 vaccine hesitancy. Despite this rationale, the literature shows contrasting results about the relationship between anxiety and vaccine acceptance in the context of the global COVID-19 pandemic. Specifically, anxiety was discussed as a potentially functional fear that predicts public health compliance behaviors [29] and correlates with the decision to accept the vaccine [30], whereas other studies showed that COVID-19-related anxiety was associated with higher vaccine acceptance [31]. Moreover, anxiety disorders were not associated with vaccine hesitancy [32] and did not predict vaccine acceptance [33,34].

Regarding pregnant and breastfeeding/puerperium women, despite the data that continue to confirm the safety of vaccines against COVID-19 [35], attitudes towards vaccination among this population show a range of vaccine hesitancy between 26% and 57% [36]. Moreover, the literature has highlighted that higher anxiety levels in pregnant women increase vaccine hesitancy [37]. Therefore, although it is well established that pregnant women have an increased risk of anxiety particularly due to concern about the fetus and their health [38], a deeper understanding of the relationship between fear and anxiety concerning COVID-19 and vaccine hesitancy in pregnant and breastfeeding women is required. Exploration of these variables will inform vaccination campaigns to help address the challenges that are still posed today by the COVID-19 pandemic [39].

The main aim of this study was to identify the determinants of COVID-19 vaccination hesitancy and anxiety levels among pregnant and breastfeeding women in Italy. This study also described the COVID-19 vaccination hesitancy and anxiety levels in the sample.

Specifically, this study addressed the following research questions.

- What is the prevalence of COVID-19 vaccination hesitancy and anxiety in Italian pregnant and breastfeeding/puerperium women?
- Is there a correlation between COVID-19 vaccination hesitancy, anxiety levels, and sociodemographic and clinical characteristics in Italian pregnant and breastfeeding/puerperium women?

## 2. Materials and Methods

### 2.1. Design

A multicenter, cross-sectional telephone survey was conducted between January 2022 and February 2022. The STrengthering the Reporting of Observational Studies in Epidemiology (STROBE) guidelines were adopted for reporting [40].

### 2.2. Study Setting, Sampling, and Inclusion Criteria

Participants were recruited through a consecutive sampling method from the maternal services of two hospitals in the center and the south of Italy during their routine obstetric visits or contacted by phone by a specialized nurse. To be enrolled, the women had to be able to understand Italian, be pregnant or going to give birth within 6 months, and have breastfed. Women at high risk during pregnancy and/or while breastfeeding, women with postpartum depression, those who had not breastfed, and those who had not agreed to sign the informed consent form were not eligible for participation.

### 2.3. Data Collection

Between January and February 2022, a research nurse administered a structured questionnaire via phone, contacting all patients who had agreed to participate and provided informed consent during their routine obstetric visits in the maternal services of the participating hospitals.

All the participants’ responses to the questionnaire were entered directly into a computer platform by setting up a specific form for data entry.

#### Data Collection and Measurements

The questionnaire used for data collection was composed of the following five sections.

(1) The first part explored demographic data (age, nationality, ethnic group, educational qualification, employment status, marital status) and clinical history (if pregnant: which week of pregnancy and any obstetric risks; state of breastfeeding: in which month after the birth and in at which gestational age the birth took. (2) The second part investigated the participant’s vaccination status during pregnancy or while breastfeeding (if vaccinated: how many doses, which type of vaccine, and any adverse events following immunization (AEFIs)) and whether participants had been infected with COVID-19 (if yes: in which period and any signs and symptoms). (3) The third part investigated why the women decided not to be vaccinated (if applicable) and where they sought information related to vaccination. (4) The fourth part evaluated the attitudes toward vaccination using the Italian version of the Vaccination Attitudes Examination (VAX) Scale [41] validated by Tomietto and colleagues [42]. The scale consists of 12 items rated on a Likert scale from one (totally disagree) to seven (totally agree) and the average is calculated to provide an overall score; the lower the scores on the VAX scale, the greater the positive attitude towards vaccination. The scale demonstrated optimal psychometric characteristics (Cronbach’s alpha = 0.89, ranging from 0.77 to 0.86), and the authorization to use the Italian version was granted by the authors. In this study, the VAX scale showed an overall Cronbach’s alpha of 0.85 and a range from 0.85 to 0.92.

(5) The last section of the instrument assessed the anxiety rates of the sample through the Self-Rating Anxiety Scale (SAS) [43], a 20-item instrument used to self-assess anxiety. Each item uses a 4-point Likert scale for responses, ranging from “almost never” to “very often.” A final score of 0 to 20 indicates a very low level of anxiety, 21–40 indicates a low level of anxiety, 41–60 means a moderate anxiety level, and 61–80 indicates a high level.

Previous researchers have tested the psychometric proprieties of the SAS and showed a Cronbach’s alpha = 0.83. In the present study, the scale presented a satisfactory reliability coefficient (Cronbach’s alpha = 0.84).

In the pilot study phase, the entire data set collected using the questionnaire was pre-tested on a sample of 35 subjects from the target group. This was useful to identify any problems with the administration of the questionnaire by phone before collecting data from the entire sample of pregnant women. In particular, the pilot phase confirmed the validity of the data collection instrument, and also allowed for the verification that the instructions for completing the instrument were clear and exhaustive and identifying the average time taken to complete the questionnaire during each telephone interview.

### 2.4. Data Analysis

Descriptive statistics were used to describe the main demographic and clinical characteristics of the sample, their attitudes towards COVID-19 vaccination, and the results of the VAX Scale questionnaire and the SAS scale. The Cronbach’s alpha was calculated to test each instrument’s reliability, overall and separately for each sub-scale domain, when possible. Values > 0.90 are considered excellent, values > 0.70 to <0.90 are rated as good, values > 0.60 to <0.70 are acceptable, and alpha values < 0.60 are not acceptable [44].

The association between demographic and clinical characteristics and the two scales was explored by computing simple regression coefficients and then fitting two multiple regression models. A priori covariates were included and tested for multicollinearity. Potential transformation, interaction, and/or quadratic/cubic terms were investigated. Due to a high degree of collinearity between COVID-19 vaccination status and the presence of adverse events following vaccination, only the latter covariate was reported in the final multivariate models. Similarly, due to a high degree of collinearity between prior SARS-CoV-2 infection and symptomatic COVID-19, only the former was kept in the final model due to a higher R2. The validity of each final regression model was assessed as follows: the assumption of constant error variance was checked graphically, plotting Pearson residuals vs. fitted values, and formally, using the Cook–Weisberg test for heteroskedasticity. High-leverage observations were identified by computing Pearson, standardized, and studentized residuals, and Cook’s D influence. In all models, we found less than 10 high-leverage observations and, after excluding these, we noted no substantial changes.

Statistical significance was defined as a two-sided *p*-value < 0.05 for all analyses, which were carried out using Stata [45].

### 2.5. Ethical Considerations

Participation in the study was completely voluntary for all participants and included compliance with the standards of informed consent, data confidentiality, and anonymity [46]. The data collection and analysis were designed to ensure data confidentiality and followed national and European laws and the Personal Data Act [47]. Administrative authorizations were obtained from the participating centers and eligible participants received details on the purpose and procedures of the study, and information on the data management. Only after giving their consent, the participants were interviewed. Information regarding the study, ethical issues, and the researcher’s contact details were also provided by phone. The electronic data were saved in a protected folder, accessible only by the principal investigator.

## 3. Results

### 3.1. Sample Characteristics

Overall, 452 women met the inclusion criteria and were invited to participate. Of these, 435 women agreed, and 420 completed the questionnaire and were included in the evaluation of vaccine hesitancy (response rate: 93%). The mean age of the participants was 33.6 years (SD = 5.4). Most participants were Italian (84.7%), married (83.9%), Caucasian (92.6%), currently employed (60.2%), had a high school education (44.4%), and were primiparous (83.3%). The sample consisted of 159 (36.6%) pregnant women and 276 (63.4%) women up to 6 months after childbirth. At delivery, 76.4% of women were between 38 and 40 weeks of pregnancy, and 17.4% were between 34 and 37 weeks; among the currently pregnant women, the gestational age in weeks was 37.1%, 35.9%, 17.6%, and 9.4% in the classes >37, 21–30, ≤12–16, and 17–20, respectively. Table 1 shows the socio-demographic characteristics of the sample and their obstetric histories.

### 3.2. COVID-19 Vaccination Status and COVID-19 Disease

Of all the respondents, 14.0% of women did not receive the COVID-19 vaccine, but 7.7% were willing to. Among the vaccinated participants (78.3%), 70.1% received their first dose after delivery, 28.7% before pregnancy, and 11.0% during pregnancy. Among the women who received the vaccine, 44.5% reported AEFIs (48.0%), pain at the injection site (44.6%), muscle pain (39.2%), fatigue (27.0%), and headache (16.2%). Approximately 37.2% of the sample reported previous contraction of COVID-19, and for 56.0% of them, the infection was contracted with signs and symptoms during pregnancy (83.7%) (Table 1).

### 3.3. Reasons to Get Vaccinates and Sources of Information

The results showed that 71.9% of the sample received a recommendation to get the COVID-19 vaccine from a general practitioner (GP) (44.0%) and/or a gynecologist (46.7%). The main reasons for vaccination were to protect the fetus from the consequences of the coronavirus disease (30.3%), trust in vaccination (29.7%), and perception of vaccines as safe tools (22.7%). The most frequent reasons mentioned for not getting vaccinated were concerns about the possible side effects including miscarriage, autism, or developmental disorders (32.6%); preference towards natural immunity (9.0%); and vaccination was not recommended by health professionals (7.9%) or relatives/friends (6.7%).

Among the 60 unvaccinated women, we found that 80% of them decided not to get vaccinated due to their convictions, while 14.2% were because of a gynecologist’s or GP’s recommendation (Table 2).

### 3.4. Vaccination Attitudes and Anxiety Levels of the Sample

The descriptive statistics of the VAX scale are reported in Table 3 and show a total mean score (SD) of 3.35 (1.6). The mean scores (SD) of the main factors of the VAX scale were as follows: “mistrust in the benefits of the vaccine”, mean = 2.6 (1.5); “worries about the future unforeseen effect”, mean = 4.8 (1.6); “concerns about commercial profiteering”, mean = 2.7 (1.6); and “preference for natural immunity”, mean = 3.3 (1.6).

Table 4 reports the descriptive statistics for the SAS scale, revealing a mean value of 39.9 (SD = 10) for the “Anxiety Index”. Most participants (n = 310, 75%) reported an anxiety index equal to or lower than the threshold, whereas overall, 25% (n = 103) of the sample ranged between “minimal to moderate” and “marked to severe” anxiety levels.

### 3.5. Relationship between Anxiety Levels, Attitude towards Vaccinations, and Characteristics of the Sample

In the final multiple regression model, vaccine hesitancy was significantly and positively associated with “adverse events after vaccination” (β = 4.20; 95% CI: 1.25, 7.15; *p* = 0.006), and negatively associated with a higher educational level (*p* < 0.05) (Table 5); potential, independent predictors of SAS levels were a higher mean age of the participant (β = 2.75; 95% CI: 0.12; 5.37; *p* = 0.041) and the employment status (β= −3.39; 95% CI: −6.25, −0.52; *p* = 0.02) (Table 6). We did not observe a significant association between higher levels of vaccine hesitancy and anxiety; both had a *p* > 0.05 (Table 5 and Table 6).

## 4. Discussion

This study aimed to evaluate the attitudes toward COVID-19 vaccination of Italian pregnant and breastfeeding women, and showed several interesting findings that expanded our understanding of SARS-CoV-2 vaccination hesitancy in this population and the variables influencing it. To date, there are still gaps in knowledge regarding which and how individual characteristics influence pregnant and breastfeeding women’s decision to get vaccinated. It is well established that improved knowledge and perceptions of the COVID-19 vaccine improve vaccination acceptance, which in turn improves vaccine uptake. However, it is still fundamental to provide evidence to determine how to improve these perceptions to avoid vaccination refusal. The results of this study provided insights regarding the determinants of vaccination in this population by considering the association between several individual, clinical, and demographic factors.

A high rate of vaccination uptake (78.3%) was found in the overall sample; the majority received their first immunization after delivery or before pregnancy, and only a small proportion (11%) received it during pregnancy. A review [48] highlighted a similar vaccination rate, ranging between 29.7% and 77.4%.

Most women decided to get vaccinated to protect their baby, and because of the trust in vaccination, which was perceived as a safe tool, in agreement with the results of Hagenbeck and colleagues [49]. Furthermore, the data suggested that GPs and gynecologists provided recommendations to inform women, but they were not the decisive reason why the women decided to get vaccinated. These results are in contrast with previous pre- [50] and post-pandemic [39] evidence where healthcare professionals’ recommendation was identified as the most important factor in maternal decision-making to get vaccinated. However, it is well established that several factors influence people’s decision-making processes about vaccination such as prior personal beliefs and the type of messages received from various sources of information, including media and social media, the community, family members, and peers [51]. In this vein, it is possible that in our sample, a positive previously formed belief regarding vaccination existed, and that this may have had a greater influence on decision-making than the healthcare professionals’ recommendations [51]. Healthcare professionals should focus on the protective role and safety aspects of being vaccinated during pregnancy and puerperium through health education interventions rather than in disseminating information about the current and updated recommendations [52].

A fear of vaccine-related side effects regarding their own and the child’s health was the most relevant factor affecting the women’s willingness to get vaccinated. This is consistent with the results of other studies reporting that among the determinants of vaccine hesitancy against COVID-19, concerns around vaccine safety [53,54] and vaccine efficacy for the immune system of fetuses/infants [39,55,56] are the main barriers.

The results from multivariate linear regression showed that previous experiences of adverse events after vaccinations are a predictor of vaccine hesitancy; meanwhile, as highlighted by previous evidence [36], a higher educational level was related to lower vaccine hesitancy [39,57,58]. The findings of this study suggest that educational level is related to the individual’s ability to understand and evaluate both risks and benefits of COVID-19 vaccination [36].

A possible effective intervention to overcome the barrier related to lower levels of knowledge of COVID-19 vaccination is establishing forums where pregnant women discuss their motivations to get vaccinated or not and provide useful and reliable information and educational content to promote vaccination [59]. However, given that social influences act as both a barrier and facilitator to vaccination depending on the nature of the experiences shared [39], such a forum should emphasis the low likelihood of severe negative experiences.

The concept of “deep belief” is also relevant to addressing future strategies and interventions to promote vaccination in this population as it may influence the choice to get vaccinated. Education delivered by healthcare professionals may assist in changing “false deep beliefs” amongst unvaccinated women. A potential solution is offered by the results of a recent study [60] that showed the effectiveness of an educational intervention in improving pregnant women’s knowledge, health beliefs, as well as self-reported compliance with preventive behaviors regarding COVID-19, which was based on the Health Belief Model (HBM). The Health Belief Model (HBM) is a large psychosocial behavior change model that includes five main structures: (i) perceived susceptibility, (ii) perceived severity, (iii) perceived benefits, (iv) perceived barriers, (v) cues to action, and (vi) self-efficacy [61]. 

The results of the SAS scale showed a normal level of anxiety in the whole sample. A small proportion reported minimal to moderate (19.9%) and marked to severe anxiety (5.1%). Older maternal age and unemployed status seem to be associated with higher levels of anxiety. These results are in line with those of Tearne and colleagues [62], suggesting that an age equal to or greater than 37 years old is a potential predictor of anxiety, depression, and stress symptoms in women. Employment status seems to be a risk factor for maternal mental health, particularly depressive symptomatology after birth [63].

In line with previous research [32,33,34], the current study found that general anxiety did not significantly predict vaccine hesitancy. Thus, in agreement with previous research, the influence of general traits or state anxiety was found to be minimal, and instead, vaccine hesitancy is dependent on the source of the anxiety or fear. 

### 4.1. Implications for Policy and Practice

These research findings have implications for developing effective interventions that could increase pregnant and breastfeeding, puerperium women’s COVID-19 vaccine acceptance level. Our study showed high levels of vaccine hesitancy related to worries about unforeseen future effects. Therefore, educational interventions delivered by health professionals should be specifically tailored and based on the HBM.

The findings of this study, combined with those of previous research, indicate that campaigns and interventions to increase vaccination should address specific anxieties around side effects, harm to the fetus, and needles, as opposed to specifically targeting populations with high levels of general anxiety [39]. Specifically, this study identified a key area to address (possible worries about unforeseen future effects) and this factor is also a major reason for concern amongst healthcare professionals [40,54,64]. Furthermore, the finding of education as a significant predictor of vaccine hesitancy indicates that future vaccination campaigns and interventions that communicate information about the vaccine should ensure that it is comprehendible to those with low literacy levels. Moreover, the results from the regression analyses suggest that previous experiences of adverse events after vaccinations and lower educational levels are predictors of higher vaccine hesitancy in pregnant women. These results could be useful in the development of a specific framework to deliver vaccine education. Specifically, healthcare providers and professionals should find a balance between the promotion of individual health, by focusing on the clinical and demographic characteristics of the individual, and public health, by considering the main determinants of vaccine hesitancy in this population.

### 4.2. Recommendations for Further Research, Strengths, and Limitations

As one of the main factors affecting women’s vaccination hesitancy is related to worries about the future unforeseen effects of vaccination, more research is needed to better explore the role of healthcare professionals and the potential of specific interventions to improve vaccination uptake. For these reasons, to separate the influence of the pandemic from attitudes towards COVID-19 vaccination, further studies should be conducted outside the pandemic period. Finally, we found that anxiety levels did not correlate with COVID-19 vaccine hesitancy. However, it would be interesting to explore the nature of this relationship in further studies by considering other variables such as the reactiveness to the uncertainties about the COVID-19 virus, and concerns, which may differentially influence decision-making in people with high levels of anxiety compared to those with low or no levels of anxiety.

Healthcare professionals should also strengthen their educational role by developing educational interventions to enable informed decisions about health/disease issues and avoid spreading conflicting information.

The results of this study disclosed relevant aspects of the perception of the COVID-19 vaccine amongst pregnant and breastfeeding women, allowing the identification of future directions for tailoring public health campaigns to increase vaccine uptake in this population.

However, this study includes several limitations that should be considered. Because of the cross-sectional study design, causal inference based on these models should be considered with caution. Furthermore, outcomes based on self-reported measures are potentially biased by misreporting, misclassification, and social desirability. Another limitation could be the geographical location of the samples included in this study. Most of the women came from two regions of Italy, and women at high risk during pregnancy/while breastfeeding or with postpartum depression were excluded; these aspects could affect the generalizability of the results.

## 5. Conclusions

Investigating the reasons that influence vaccine hesitancy is relevant to developing targeted health policy strategies and COVID-19 vaccination programs. Health education programs that are accessible to those of low educational attainment are needed to address vaccine hesitancy, reduce the knowledge gap, and improve COVID-19 vaccination acceptance. Such educational interventions should be based on the HBM, by acting on main key factors that influence health behaviors such as an individual’s perceived threat to sickness or disease (perceived susceptibility), perceived consequences (perceived severity), potential positive benefits of action (perceived benefits), perceived barriers to action, exposure to factors that prompt action (cues to action), and confidence in ability to succeed (self-efficacy).

## Figures and Tables

**Table 1:** Overall characteristics of the sample (n = 435).

Variable	
Mean age (SD)	33.6 (5.4)
Region, %	
- Puglia	64.4
- Marche	25.5
- Abruzzo	2.5
- Other	7.6
Caucasian, %	92.6
African American, %	1.1
Hispanic/Latinx, %	1.8
Asian, %	2.3
Other, %	2.2
Educational level, %	
- Primary/lower secondary school	11.0
- High school	44.6
- Bachelor/higher	44.4
Currently employed, %	60.2
Currently not employed, %	39.8
Pregnancy status, %	
- Currently pregnant	36.6
- Puerperium/lactation	63.4
Gestational age class in weeks among currently pregnant women, %	(n = 159)
- ≤12–16	17.6
- 17–20	9.4
- 21–30	35.9
- ≥37	37.1
High-risk pregnancy, %	4.4
Low-risk pregnancy, %	8
No-risk pregnancy, %	27.1
I prefer not to answer, %	60.5
Gestational age at delivery in weeks, %	(n = 276)
- 25–33	6.2
- 34–37	17.4
- 38–40	76.4
Primiparous/multiparous women, %	51.7
Parity status, %	(n = 210)
- Primiparous	83.3
- Multiparous	16.7
Previous SARS-CoV-2 infection, %	
- Yes	37.2
- No	62.8
Time of infection, %	(n = 159)
- Before pregnancy	15.1
- During pregnancy	56.0
- During puerperium/lactation	15.1
- After puerperium/lactation	13.8
Reported symptomatic COVID-19 among the infected, %	83.7
Type of symptom(s) reported, % ^A^	
- Fever	57.9
- Muscle pain	45.9
- Cough	42.1
- Smell and taste disorders	32.3
- Fatigue	22.6
- Headache	21.8
- Other ^B^	21.2
SARS-CoV-2 vaccination status, %	(n = 428)
- Vaccinated	78.3
- Unvaccinated	14.0
- Willing to	7.7
Time of the first immunization, %	(n = 335)
- Before pregnancy	28.7
- During pregnancy	11.0
- After delivery	60.3
AEFIs, %	44.5
Type of AEFI reported, % ^A^	(n = 149)
- Fever	48.0
- Injection site pain	44.6
- Muscle pain	39.2
- Fatigue	27.0
- Headache	16.2
- Others ^B^	15.7

^A^ More than one answer is possible. ^B^ Includes sore throat, thoracic pain, respiratory or gastrointestinal disorders, rash, and dizziness. AEFIs: adverse events following immunization.

**Table 2:** Reasons to get the SARS-CoV-2 vaccine and sources of information: results of the questionnaire in the sample of women who responded to the questionnaire (n = 420).

Variable	
Recommended SARS-CoV-2 vaccination, %	71.9
Who recommended SARS-CoV-2 vaccination, %	(n = 302)
- General practitioner	44.0
- Gynecologist	46.7
- Other (nurse, family/friends, mass-media)	9.3
Who recommended not to get SARS-CoV-2 vaccine, % ^A^	(n = 60)
- I decided by myself	80.4
- General practitioner/gynecologist	14.2
- Other (nurse, family/friends, mass media)	5.4
Main reasons to get vaccinated, %	(n = 330)
- Recommended by healthcare professionals	3.3
- Perception of COVID-19 as a very serious disease	4.2
- Fear of serious consequences of COVID-19	4.9
- Protecting the fetus	30.3
- Trust in vaccinations	29.7
- Perception of vaccines as a safe tool	22.7
- Other, minor reasons	4.9
Main reasons not to get vaccinated, %	(n = 89)
- Not recommended by healthcare professionals	7.9
- Not recommended by relatives/friends	6.7
- Contrasting information about vaccines	4.5
- Perception of COVID-19 as not serious	3.4
- No fear of serious consequences of COVID-19 on the fetus	1.1
- Perception of vaccines as unsafe tools	4.5
- Fear of vaccine-related SAEs	32.6
- Preference towards natural immunity	9.0
- Other, minor reasons	3.3

^A^ Among the 60 unvaccinated women.

**Table 3:** Results of the VAX Scale questionnaire among the selected sample (N = 415).

Variable	Mean (SD)	Positive Answers (95% CI), % *
A. “Mistrust in the benefits of the vaccine” factor (α = 0.92)	2.6 (1.5)	
1. I feel unsafe after being vaccinated	2.8 (1.6)	17.6 (9.8–28.5)
2. I cannot rely on vaccines to stop serious infectious diseases	2.3 (1.4)	9.1 (1.9–24.3)
3. I do not feel protected after getting vaccinated	2.8 (1.6)	15.7 (7.6–26.5)
B. “Worries about the future unforeseen effect” factor (α = 0.85)	4.8 (1.6)	
4. Although most vaccines appear to be safe, there may be problems that we have not yet discovered	5.2 (1.6)	74.7 (69.6–79.6)
5. Vaccines can cause unforeseen problems in children	4.5 (1.7)	53.7 (47.0–60.5)
6. I worry about the unknown effects of vaccines in the future	4.8 (1.7)	65.8 (60.0–71.5)
C. “Concerns about commercial profiteering” factor (α = 0.89)	2.7 (1.6)	
7. Vaccines make a lot of money for pharmaceutical companies, but do not do much for regular people	3.0 (1.8)	24.6 (16.5–34.0)
8. Authorities promote vaccination for financial gain, not for people’s health	2.8 (1.7)	20.0 (12.4–30.8)
9. Vaccination programs are a big con	2.4 (1.5)	13.3 (5.3–24.5)
D. “Preference for natural immunity” factor (α = 0.92)	3.3 (1.6)	
10. Natural immunity lasts longer than a vaccination	3.4 (1.6)	17.8 (9.7–28.2)
11. Natural exposure to viruses and germs gives the safest protection	3.4 (1.6)	18.1 (10.6–29.3)
12. Being exposed to diseases naturally is safer for the immune system than being exposes through vaccination	3.3 (1.5)	16.4 (8.4–27.1)
Overall scale reliability coefficient (α = 0.84)		

* Subjects who rated each item with a score ≥5 on a 7-point Likert scale, where 1 = strong disagreement and 7 = strong agreement. α = Cronbach alpha, assessed separately for each scale’s domain.

**Table 4:** Results of the Zung Anxiety Self-Assessment Scale (SAS) among the selected sample (N = 413).

Items	Positive Answers (95% CI), % *
1. I feel more nervous and anxious than usual	19.1
2. I feel afraid for no reason at all	11.2
3. I get upset easily or feel panicky	8.0
4. I feel like I’m falling apart and going to pieces	5.1
5. I feel that everything is all right and nothing bad will happen	52.3
6. My arms and legs shake and tremble	5.4
7. I am bothered by headaches, neck, and back pains	9.2
8. I feel weak and get tired easily	17.9
9. I feel calm and can sit still easily	31.4
10. I can feel my heart beating fast	10.0
11. I am bothered by dizzy spells	4.1
12. I have fainting spells or feel faint	3.1
13. I can breathe in and out easily	14.8
14. I get feelings of numbness and tingling in my fingers and toes	4.8
15. I am bothered by stomach-aches or indigestion	10.1
16. I have to empty my bladder often	29.3
17. My hands are usually dry and warm	33.9
18. My face gets hot and blushes	9.7
19. I fall asleep easily and get a good night’s rest	51.6
20. I have nightmares	4.5
Overall scale reliability coefficient (α = 0.84)	
Anxiety Index: **	
Mean value (SD)	39.9 (10.0)
By score categories, % (n)	
- Below 45 (normal)	75.0 (310)
- 45–59 (minimal to moderate anxiety)	19.9 (82)
- 60–74 (marked to severe anxiety)	5.1 (21)
- ≥75 (most extreme anxiety)	0.0

* Subjects who answered each item with a score = 3 (“good part of the time”) or score = 4 (“most/all of the time”) on a 4-point Likert scale. ** Computed by adding up the total SAS raw score and re-coding each total score to the corresponding Anxiety Index. α = Cronbach alpha.

**Table 5:** Relationship between the VAX Scale and selected maternal and gestational characteristics.

Variable	Raw Coeff. (95% CI)	*p*	Adjusted Coeff. * (95% CI)	*p*
Age, 10-year increase	−1.39 (−3.70; 0.91)	0.2	0.42 (−2.67; 3.51)	0.8
Educational level, 1-category increase				
- Primary/lower secondary school	0 (ref. cat.)	--	0 (ref. cat.)	--
- High school	−5.76 (−9.86; −1.69)	0.006	−5.42 (−10.6; −0.25)	0.040
- Bachelor/higher	−9.97 (−14.0; −5.89)	<0.001	−7.22 (−12.6; −1.81)	0.009
Employed, yes vs. no	−3.84 (−6.33; −1.34)	0.003	−1.35 (−4.73; 1.02)	0.4
Married/cohabiting vs. single/separated/divorced	4.03 (−0.77; 8.84)	0.09	3.72 (−4.13; 11.6)	0.4
Gestational age at delivery, 1-week increase	0.90 (−1.76; 3.55)	0.5	2.73 (−0.25; 2.71)	0.07
Prior birth, yes vs. no	2.43 (−0.05; 4.90)	0.054	−0.05 (−3.08; 2.98)	0.9
SARS-CoV-2 vaccination, yes vs. no	−2.88 (−5.68; −0.06)	0.045	--	--
AEFIs, yes vs. no	3.55 (1.01; 6.09)	0.006	4.20 (1.25; 7.15)	0.006
Prior SARS-CoV-2 infection, yes vs. no	3.81 (1.24; 6.40)	0.004	2.97 (−0.18; 6.13)	0.065
Symptomatic COVID-19, yes vs. no	−0.81 (−7.86; 6.23)	0.8	--	--
Recommended SARS-CoV-2 vaccination, yes vs. no	−2.50 (−5.32; 0.33)	0.083	−1.71 (−5.20; 1.79)	0.3
Total SAS Scale score, 10-point increase	−0.02 (−1.27; 1.23)	0.9	0.34 (−1.28; 1.96)	0.7

Coeff.: coefficient; CI: confidence interval; ref. cat.: reference category; AEFIs: adverse events following immunization; SAS: Zung Anxiety Self-Assessment Scale. * Multivariate linear regression including 213 observations. Due to a high degree of collinearity between SARS-CoV-2 vaccination status and the presence of AEs following vaccination, only the latter covariate was reported in the final multivariate model due to a higher R2. Similarly, given the high degree of collinearity between prior SARS-CoV-2 infection and symptomatic COVID-19, only the former was kept in the final model.

**Table 6:** Relationship between the Zung Anxiety Self-Assessment Scale (SAS) and selected maternal and gestational characteristics.

Variable	Raw Coeff. (95% CI)	*p*	Adjusted Coeff. * (95% CI)	*p*
Age, 10-year increase	−2.51 (−4.30; −0.72)	0.006	2.75 (0.12; 5.37)	0.041
Educational level, 1-category increase				
- Primary/lower secondary school	0 (ref. cat.)	--	0 (ref. cat.)	--
- High school	−1.89 (−5.14; 8.37)	0.3	−3.61 (−8.07; 0.85)	0.11
- Bachelor or higher	−4.28 (−7.53; −1.03)	0.010	−4.56 (−9.24; 0.12)	0.056
Employed, yes vs. no	−4.29 (−6.23; −2.34)	<0.001	−3.39 (−6.25; −0.52)	0.02
Married/cohabiting vs. single/separated/divorced	−9.85 (−13.5; −6.17)	<0.001	−2.73 (−9.47; 4.02)	0.4
Gestational age at delivery, 1-week increase	−1.39 (−3.42; 0.66)	0.18	−0.09 (−2.67; 2.48)	0.9
Prior birth, yes vs. no	0.03 (−1.91; 1.97)	0.9	1.56 (−1.03; 4.15)	0.2
SARS-CoV-2 vaccination, yes vs. no	−1.02 (−3.16; 1.12)	0.3	--	--
AEFIs, yes vs. no	−0.61 (−2.87; 1.65)	0.6	0.17 (−2.41; 2.76)	0.9
Prior SARS-CoV-2 infection, yes vs. no	2.51 (0.51; 4.50)	0.014	2.08 (−0.63; 4.81)	0.13
Symptomatic COVID-19, yes vs. no	−4.56 (−8.78; −0.33)	0.035	--	--
Recommended SARS-CoV-2 vaccination, yes vs. no	−1.60 (−3.76; 0.56)	0.15	−2.53 (−5.51; 0.46)	0.10
Total VAX Scale score, 10-point increase	−0.02 (−0.88; 0.84)	0.9	0.02 (−0.09; 0.14)	0.7

Coeff.: coefficient; CI: confidence interval; ref. cat.: reference category; AEFIs: adverse events following immunization. * Multivariate linear regression including 213 observations. Due to a high degree of collinearity between SARS-CoV-2 vaccination status and the presence of AEs following vaccination, only the latter covariate was reported in the final multivariate model. Similarly, due to a high degree of collinearity between prior SARS-CoV-2 infection and symptomatic COVID-19, only the former was kept in the final model due to a higher R2.

## Data Availability

The data presented in this study are available on request from the corresponding author.
